# Pathways with PathWhiz

**DOI:** 10.1093/nar/gkv399

**Published:** 2015-05-01

**Authors:** Allison Pon, Timothy Jewison, Yilu Su, Yongjie Liang, Craig Knox, Adam Maciejewski, Michael Wilson, David S. Wishart

**Affiliations:** 1 Department of Computing Science, University of Alberta, Edmonton, Alberta, T6G 2E8, Canada; 2 Department of Biological Science, University of Alberta, Edmonton, Alberta, T6G 2E8, Canada; 3 National Institute for Nanotechnology, 11421 Saskatchewan Drive, Edmonton, Alberta, T6G 2M9, Canada

## Abstract

PathWhiz (http://smpdb.ca/pathwhiz) is a web server designed to create colourful, visually pleasing and biologically accurate pathway diagrams that are both machine-readable and interactive. As a web server, PathWhiz is accessible from almost any place and compatible with essentially any operating system. It also houses a public library of pathways and pathway components that can be easily viewed and expanded upon by its users. PathWhiz allows users to readily generate biologically complex pathways by using a specially designed drawing palette to quickly render metabolites (including automated structure generation), proteins (including quaternary structures, covalent modifications and cofactors), nucleic acids, membranes, subcellular structures, cells, tissues and organs. Both small-molecule and protein/gene pathways can be constructed by combining multiple pathway processes such as reactions, interactions, binding events and transport activities. PathWhiz's pathway replication and propagation functions allow for existing pathways to be used to create new pathways or for existing pathways to be automatically propagated across species. PathWhiz pathways can be saved in BioPAX, SBGN-ML and SBML data exchange formats, as well as PNG, PWML, HTML image map or SVG images that can be viewed offline or explored using PathWhiz's interactive viewer. PathWhiz has been used to generate over 700 pathway diagrams for a number of popular databases including HMDB, DrugBank and SMPDB.

## INTRODUCTION

Pathway diagrams are the road maps of biology. They have long been used as visual tools to map out complicated biological processes over space and time. In their simplest form, pathway diagrams can be used to illustrate the connections between genes, proteins and/or metabolites at the cellular or subcellular level. More complicated pathway diagrams attempt to extend these connections to tissues, organs or entire organisms, thereby adding even greater biological context. Regardless of whether the actual pathways are simple or complicated, the goal of a pathway illustration is to render complex biological processes and connections in a way that a viewer can easily understand. There is no single correct way to illustrate a biological pathway. Indeed, the level of detail and the amount of information conveyed for identical biological pathways can vary tremendously.

These differences often depend on the intended purpose or the state of knowledge of a given pathway. Certainly when pathway diagrams are created for textbooks or wall charts, the pathway illustrator is expected to generate complex, richly detailed, colourful works of art (1). In these artistic renderings the structures of metabolites, proteins, DNA, cell membranes, cell structures, cell organelles and even relevant organs are often shown along with labels, directional arrows and detailed notes or commentaries. However, when pathway diagrams are generated for internet applications, this kind of artistry (and much of the biological context) is typically sacrificed in favour of generating machine-readable wire diagrams or schematics. The most appealing feature of these simplified internet pathway diagrams is that they are often richly hyperlinked and highly interactive. Many popular web-based pathway databases, such as KEGG (2,3), MetaCyc (4), Wikipathways (5) and Reactome (6,7) contain these kinds of simplified, web-accessible pathway diagrams.

More recently, a number of these pathway databases have made their software available to allow others to illustrate or generate web-compatible pathway diagrams. In particular, BioCyc's Pathway Tools (8), Wikipathway's PathVisio software (9) and VANTED (10) let users generate, analyse and share biological pathways using platform-specific, downloadable software. These downloadable programs support the generation of pathways in machine-readable formats such as BioPAX, with some even offering online wiki-style tools for collaborative pathway generation (9). However, because the focus of these programs is primarily on web compatibility, the pathway diagrams tend to be quite basic, mainly consisting of lines, text and simple shapes with minimal to no colouring. In addition to these pathway-rendering tools for web-viewable pathways, there are also a number of excellent platform-specific software packages for generating ‘internally readable’ biological pathways or network diagrams including Cytoscape (11), CellDesigner (12), PathCase (13) and VisANT (14). However, just as with the aforementioned programs, these tools tend to generate very simplified, highly schematic diagrams that tend to be more pleasing to computers than to the human eye.

Ideally what is needed is a freely available, user-friendly, platform-independent system (i.e. a web server) that provides the capacity to generate colourful, visually pleasing and biologically complete pathway diagrams like those found in textbooks while at the same time supporting the generation of machine-readable, interactive, richly hyperlinked pathway diagrams that are compatible with modern web browsers. The fact that no such software system exists led us to develop a novel concept for pathway illustration: a pathway-drawing web server called PathWhiz.

PathWhiz is essentially a web server designed for the facile creation of colourful, visually pleasing and biologically accurate pathway diagrams that are machine-readable, interactive and fully web compatible. PathWhiz differs from other pathway drawing tools in that it is a web server rather than a stand-alone program. Therefore, unlike most downloadable software programs, PathWhiz is platform independent. This makes PathWhiz accessible from almost any place and compatible with essentially any operating system. In addition, its web presence allows PathWhiz to house a public library of pathways and pathway components that can be easily viewed, used, manipulated and constantly expanded upon by its users. PathWhiz also differs from most other pathway drawing tools with regard to the level of biological detail, physiological context and biological complexity that it can support. This is because it uses a specially designed drawing palette to render metabolites (including automated structure generation), proteins (including quaternary structures, covalent modifications and cofactors), nucleic acids, membranes, subcellular structures, cells, tissues and organs. Both small-molecule and protein/gene pathways can be constructed using a simple graphical interface to combine specific pathway processes such as reactions, interactions, binding events and transport actions. Pathways drawn in PathWhiz can be saved in machine-readable BioPAX, SBGN-ML (Systems Biology Graphical Notation Markup Language) and SBML (Systems Biology Markup Language) (15–17) formats, or they can be saved in PathWhiz's custom PWML (Pathway Markup Language) data format or as PNG (Portable Network Graphic), HTML image map or SVG (Support Vector Graphic) images that can be viewed offline or explored using PathWhiz's interactive viewer. Additional details and specific examples regarding the design and implementation of PathWhiz are discussed below.

## IMPLEMENTATION

PathWhiz consists of three major components: the Pathway Editor, the Pathway Viewer and the PathWhiz Data Repository for capturing metadata about each pathway and pathway object or process. Pathways are generated and edited using the Pathway Editor while the Pathway Viewer is for visualizing, printing and downloading the finished pathways. PathWhiz was built on a Ruby on Rails (http://rubyonrails.org, version 4.2.0) web framework incorporating a MySQL relational database (https://www.mysql.com, version 5.1.50) to manage all of the pathway data, including entity relationships, external references, descriptions, visualization specifications and chemical structures. The front-end web client is controlled by Ruby on Rails combined with Backbone.js (http://backbonejs.org, version 1.0.0) as the front-end web framework for the editor. PathWhiz collects and manages all the data necessary to produce PNG and SVG images, as well as BioPAX (15), SBGN-ML (16), SBML (17) and PWML representations of the pathway data. Image generation uses the PhantomJS WebKit (http://phantomjs.org, version 1.9.8), while custom Java plugins have been developed for BioPAX, SBGN-ML and SBML generation using the Paxtools (18), JSBML (19) and LibSBGN (http://www.sbgn.org/LibSBGN, version: Milestone 2) libraries, respectively. PathWhiz has been tested and found to be compatible with most modern web browsers including Google Chrome (v. 31 and above), Internet Explorer (v. 9 and above), Safari (v. 7 and above), Opera (v. 15 and above) and Firefox (v. 23 and above).

## PATHWAY GENERATION AND THE PATHWAY EDITOR

### Guest accounts versus private accounts

PathWhiz can be accessed either as a guest user or by creating a ‘private’ user account. The functionality of the two account types is essentially identical, with both guest users and ‘private’ users having access to the exact same editing capabilities and pathway or pathway component libraries. The main difference is that the content created by a guest user can be edited by all other guest users, while the content created by a ‘private’ user can only be edited by the account holder. The guest model for PathWhiz is akin to that of Wikipedia (http://www.wikipedia.org)—guest users are anonymous, and anyone can add a pathway or edit an existing pathway. To prevent guest pathway diagrams from being corrupted or vandalized, pathways have a ‘lock’ functionality that allows guest users to lock (or prevent) future editing of pathways by other guest users. Once a pathway is locked, it cannot be unlocked.

A locked pathway can still be selected by another user for replication (pathway replication is described in further detail later), which allows any guest user to duplicate that pathway, edit it and to save it as either a locked or unlocked pathway under a different title. This replication function allows existing pathways to be edited or altered by anyone without damaging the original pathway. Unlike guest-drawn pathways, pathway diagrams created by a user with his/her own account can only be edited by that account holder. These pathways can still be viewed and replicated by others if the user makes them publically available (which is strongly encouraged), however, they also can be made private, meaning no other users will be able to view them. This private user account option on PathWhiz allows individuals to create and save pathways that they may not wish to have public (due to publication issues or privacy concerns), to create private collections of pathways for their own use or to satisfy the need of some users to have appropriate attribution if they choose to make their personal pathway diagrams public. The developers of PathWhiz respect user privacy. Private pathway data will not be altered by anyone, including the PathWhiz web server administrators, without user permission, for as long as the web server is operational and as long as the user indicates the pathway data should remain private.

### The pathway model and metadata

**Figure 1.**
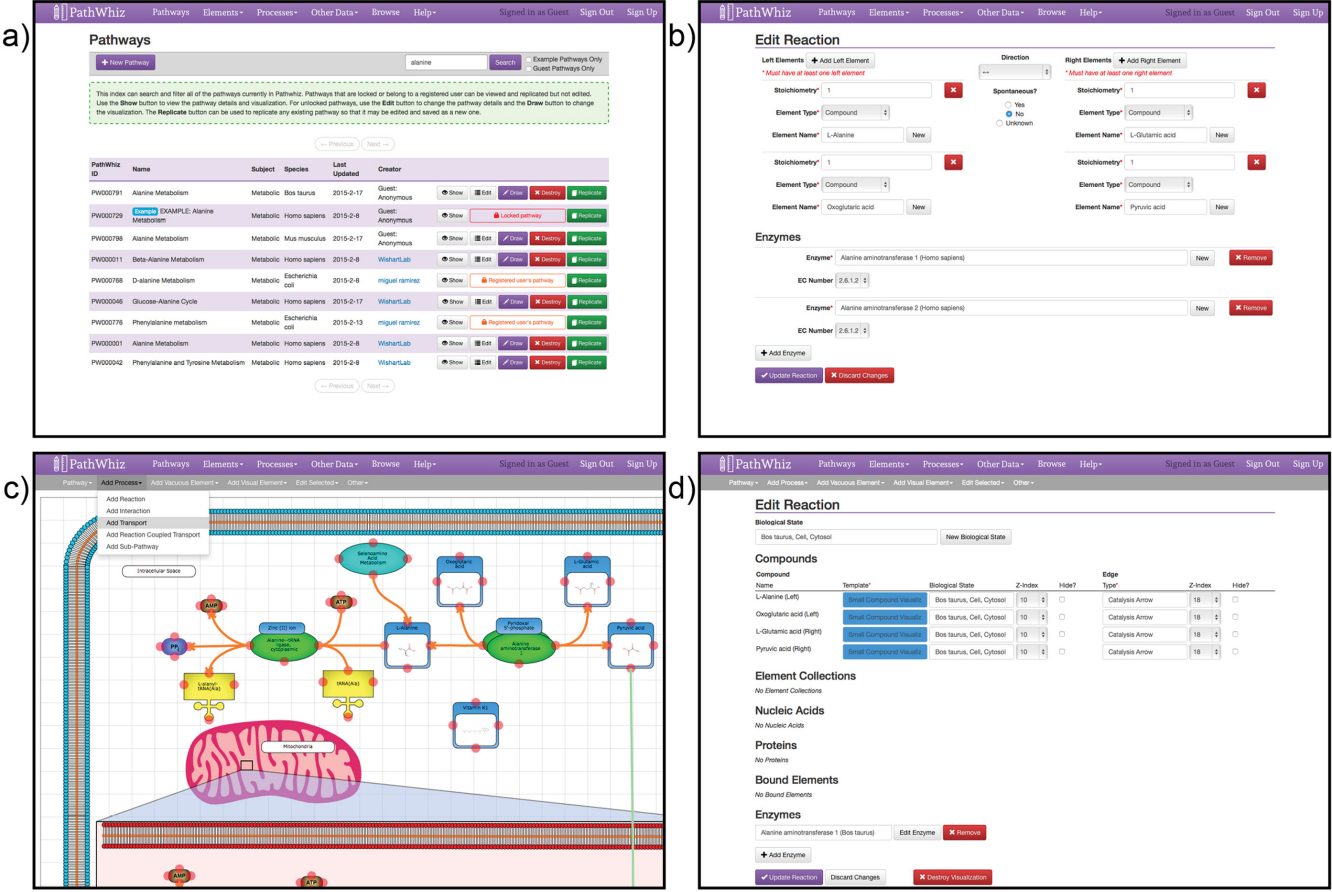
Components of the PathWhiz Editor: (**a**) browsing the pathways currently in PathWhiz, (**b**) entering pathway model metadata into the database, (**c**) working with the pathway drawing system to build a diagram and (**d**) customizing visualization attributes, such as templates and colours.

In PathWhiz, pathways are essentially stored and represented as two parts: a pathway model and a pathway visualization. Pathway models use two standard components: biological elements and biological processes, each of which is described by a specific set of metadata. Element types consist of metabolites, proteins/enzymes, cofactors, nucleic acids and biological states, while processes consist of reactions, interactions, binding events and transport activities that reference the aforementioned elements. The element and process metadata includes information such as names, accession numbers, structures, sequences, external references, cardinality, directionality, biological locations and other kinds of notes or annotations. Both elements and processes can be entered into and accessed from the public PathWhiz database using the Pathway Editor (Figure 1a and b). Data are entered using descriptive web forms, which capture all of the necessary metadata needed to describe and annotate a pathway. Though PathWhiz has the capacity to contain very detailed information about each component, only the basics are required for visualization, if preferred. Once entered into the database, elements and processes can be incorporated into any new or existing pathway. Because PathWhiz was used to generate pathway diagrams for the Small Molecule Pathway Database (SMPDB) (20), it already contains a sizeable library of elements and processes, including over 40,000 compounds from the Human Metabolome Database (HMDB) (21) and DrugBank (22), as well as over 7000 proteins from the UniProt Knowledgebase (UniProtKB) (23,24).

### Pathway visualization

Once entered into the database, pathway elements and pathway processes can be added to a pathway model and rendered in the Pathway Editor's visualization module. This pathway drawing system is a specially designed, online graphical workspace where pathway components can be rendered and manipulated to create the desired pathway visualization (Figure 1c and d). Pathway element and pathway process types are selected from the drawing system's pull-down menus and may be rendered as stand-alone entities or added to existing visualization components (e.g. connecting a series of reactions together). Rendering can be done automatically by PathWhiz (horizontally or vertically) and/or manually via the standard ‘click-and-drag’ selection and positioning mechanisms. Component positioning, layering (z-index) and connectivity are flexible and fully controlled by the user, while some component types may also be rotated and scaled. PathWhiz offers automatic saving that can be turned on or off. Each of the rendered components can be customized from a standard set of templates specific to the pathway component type, including intuitive representations of various component characteristics, such as color-coded arrows for different processes and different shapes for depicting different protein functions (e.g. enzymes versus transporters versus receptors). The full list of available templates can be seen in the pathway legend (http://smpdb.ca/pathwhiz/legend). If the appropriate metadata is available in the database, PathWhiz can also automatically render chemical structures for small-molecule compounds, choose appropriate subunit structure templates for proteins and naively arrange the elements in protein complexes.

**Figure 2.**
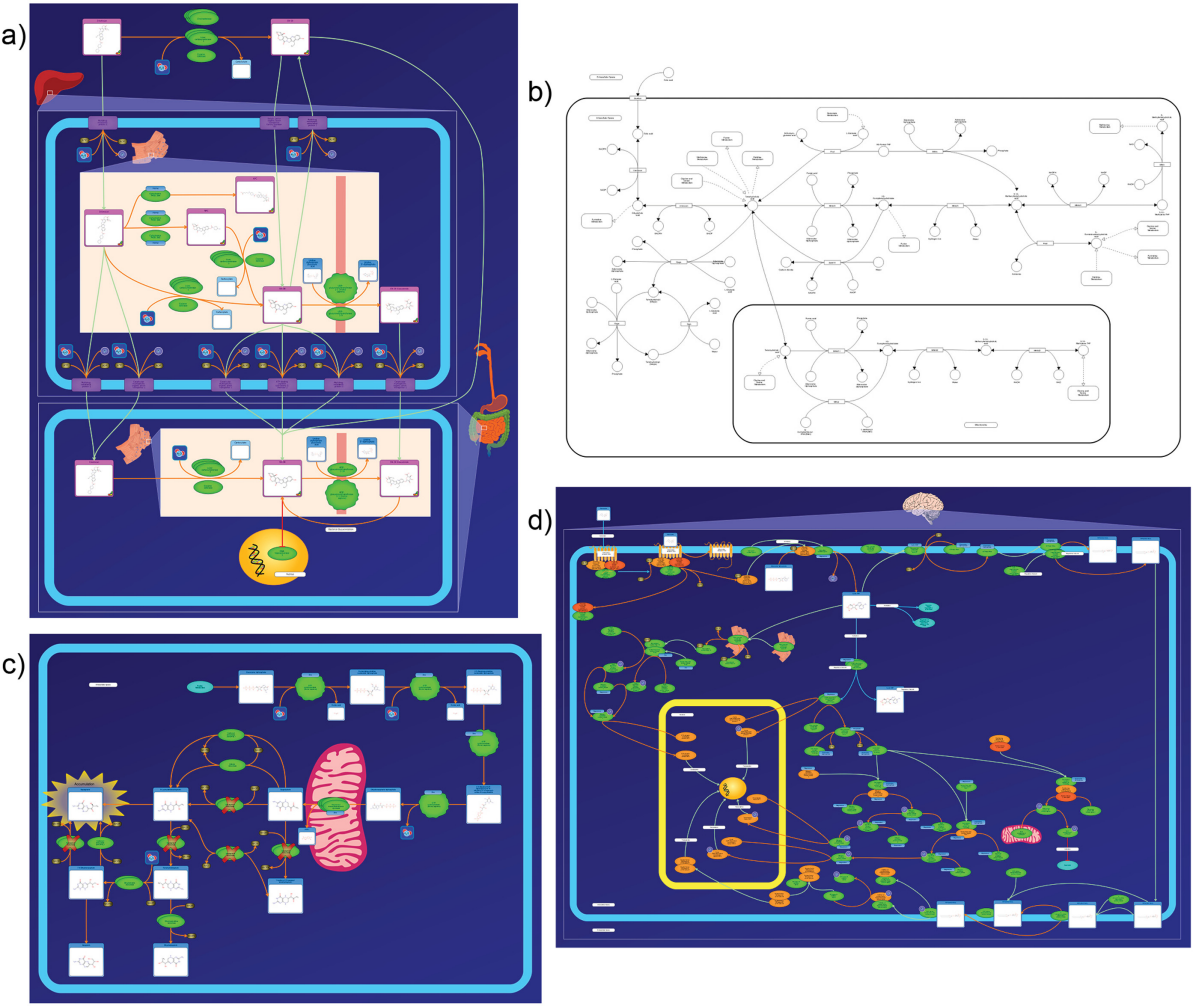
Examples of the different types of pathway diagrams that PathWhiz can produce: (**a**) a detailed small-molecule pathway (Irinotecan Metabolism in *Homo sapiens*), (**b**) a simplified pathway (Folate Metabolism in *Mus musculus*), (**c**) a disease pathway (Sepiapterin Reductase Deficiency in *Homo sapiens*) and (**d**) a protein–protein signalling pathway (intracellular signalling through adenosine receptor A2a and adenosine in *Homo sapiens*).

The PathWhiz drawing system also contains a membrane tool that allows the free-form rendering of simple or complex membranes colour-coded to indicate different membrane types. Pathways can be further augmented with the drawing system's library of stock images, which correspond to other kinds of biological elements such as organs, cell organelles, or biological ‘states’ such as mutated proteins. There are also non-biological visualization components that can be utilized to aid pathway depiction, including customizable labels, lines, and zoom boxes. In effect, the large variety of visualization options available in the drawing system's library allows the generation of pathway representations from very simple, black and white wire depictions to very detailed, colourful and image-rich diagrams (Figure 2). Quick start and detailed user manuals for the PathWhiz Editor are available online (http://smpdb.ca/pathwhiz/guides). Once a pathway diagram is completed it can be exported to various graphical and data exchange formats, as well as viewed in the interactive Pathway Viewer, which is described later.

### Replication and propagation

The Pathway Editor also contains pathway replication and pathway propagation functionality. The replication function essentially allows users to take any existing public pathway (including those that are locked or those that belong to ‘private’ users) and duplicate it so that it may be altered and/or combined with other visualizations and saved as a new pathway. Pathway replication can be done both at the start of pathway creation (i.e. a new pathway can be generated using an existing pathway as a starting template) as well as at any point during the pathway drawing process (i.e. an existing pathway can be copied/imported into the current pathway). Replication allows users to alter and append existing pathways without having to redraw complex pathways that have already been depicted by other users.

Pathway propagation is an extension of pathway replication and was developed to allow pathways to be replicated and mapped to other species (where protein names or accession numbers are different). Pathway propagation is performed using the same mechanisms as pathway replication. If the species of the new pathway is not the same as the species of the original pathway, PathWhiz automatically converts any proteins in the original pathway to the proteins corresponding to the new species. This is done using the Basic Local Alignment Search Tool (BLAST) (25) on UniProtKB (24) to look for suitable protein homologues. Proteins with matching species and expectation values (E-value) less than the user-specified cut-off are considered homologues, and the best match is chosen. If no proteins are found, PathWhiz will mark that protein as ‘Unknown’ in the newly replicated pathway, and the user can then choose to keep, change or remove it. Pathway propagation can also be done as an independent function, accessible when selecting any public pathway in the PathWhiz index. If an existing pathway is propagated in this way, multiple organisms can be selected for propagation and PathWhiz automatically fills in the details for each newly propagated pathway.

## THE PATHWAY VIEWER

**Figure 3.**
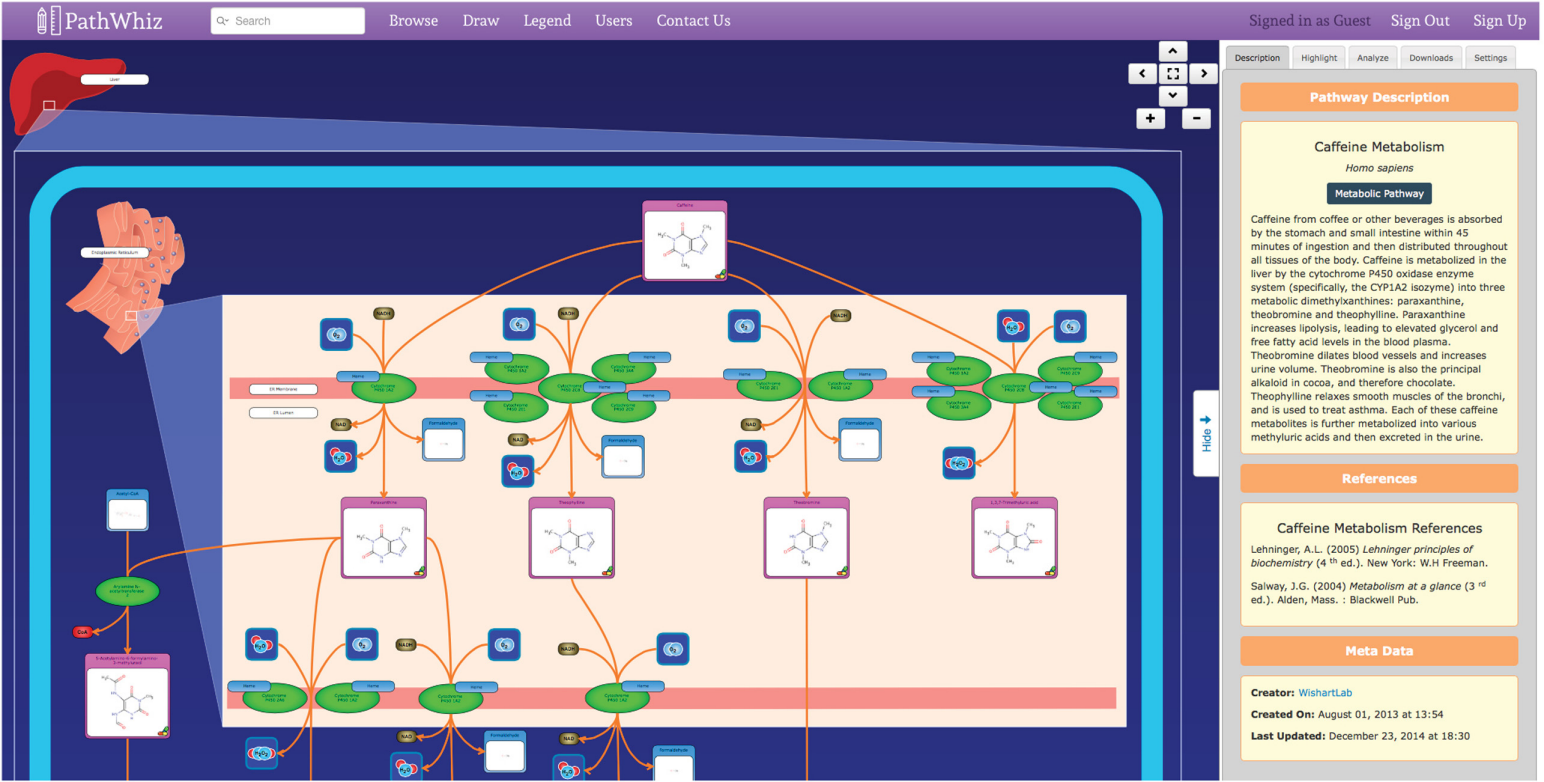
The Pathway Viewer, where pathways and pathway components can be zoomed, scrolled and highlighted. Elements are also hyperlinked for further information.

PathWhiz's Pathway Viewer (Figure 3) provides a convenient and interactive interface for viewing PathWhiz pathway diagrams. Using this viewer, a pathway diagram can be easily zoomed and navigated in a similar fashion to Google Maps (https://www.google.ca/maps). This is done using standard navigation buttons in addition to conventional mouse-activated click, drag and scroll operations. We believe this type of image navigation is very appropriate for viewing biological pathways since they are essentially road maps for biologists. The side bar of the PathWhiz Pathway Viewer provides a highlighting ability, allowing users to quickly find and jump to specific entities of interest. The side bar also provides simple analysis functionality, where users may annotate and visualize experimental metabolomic or proteomic data by mapping metabolite/protein concentrations to a colour gradient that is applied to the annotated elements. Also found on the side bar are visualization options (membrane complexity and background colour) as well as download links to the different data formats, which are described below. If the appropriate metadata is available, pathway elements in the Pathway Viewer are hyperlinked and when clicked will provide the user with a pop-up window that gives a synoptic view of the selected element, including its participating processes, biological state information and external database links.

### Data exchange formats

Through the Pathway Viewer, users may download pathway diagrams in a variety of formats for further offline use. In particular, pathway diagrams can be downloaded as SVG and PNG images, as well as in BioPAX (15) SBGN-ML (16) and SBML (17) data exchange formats. The graphical SVG and PNG images are typically intended for printing, presentations or publications, whereas BioPAX, SBGN-ML and SBML downloads contain only text data and are mainly for analysis by other software programs. The data specifications and formats used by pathway models in PathWhiz are designed to meet the standard requirements for BioPAX 3.0 and SBML Level 3, as these data exchange formats are developed specifically for sharing biological pathway data (15,17). Though the components of the PathWhiz pathway model do not map exactly to the entirety of the data representation available in BioPAX and SBML, they cover essentially all of what is required to represent most kinds of pathways (metabolic, signalling, gene and protein–protein interaction) with their organ, tissue and subcellular specificity.

As BioPAX and SBML are (currently) only able to store pathway model data and not visualization information, these formats hold detailed metadata for pathway elements and processes but lose their layout information in the conversion. Conversely, as SBGN-ML is the markup language for SBGN, a standardized graphical notation (16), it retains the pathway visualization information (including element sizes and layout) but loses much of the metadata in the conversion. While all three of these data exchange formats have their merits and uses, none are currently able to store the combination of both visual information and metadata that is contained in a PathWhiz pathway. This makes it difficult to import data in these exchange formats into PathWhiz, because much information would have to be inferred (i.e. layout) or would be missing from the imported pathway (i.e. structures, standard names and identification numbers), resulting in the addition of improperly annotated components to the database. It is for this reason that we have developed our own data exchange format, Pathway Markup Language (PWML). PWML is an XML-style representation of a PathWhiz pathway that is capable of storing both metadata and visualization information. As such, the PathWhiz Editor is capable of both exporting and importing PWML files for viewing and editing.

## DISCUSSION

Pathway diagrams drawn by skilled artists are typically the most aesthetically pleasing and easily understood of all forms of pathway visualizations. Hand-drawn pathways give the illustrator/scientist tremendous freedom to represent a pathway in a way that best communicates their understanding of the biology and the biological process being depicted. However, if one is interested in drawing dozens or hundreds of pathways or if one wants pathway illustrations to be compatible with the internet (i.e. resizable, navigable, hyperlinked) hand drawn pathways can be problematic. In particular, relying on a single artist or giving one or more pathway illustrators complete artistic freedom can lead to significant problems with productivity, internet compatibility, quality, consistency and interpretability. These problems exist because drawing pathways is just as much an art form as it is scientific pursuit, and each artist has his or her own style; that style can change or develop over time and even vary depending on the subject. Certainly many problems with consistency can be mitigated by the use of standard operating procedures (SOPs), but even SOPs will not eliminate the need for external reviewers and editors to ensure that pathways meet quality specifications.

These problems are not unique to hand-drawn pathway databases. Issues of quality, consistency and completeness seem to plague even the best internet pathway databases, even with their simplified wire-frame schematic diagrams. Nevertheless, these were precisely the problems we experienced when we first began development of a pathway database called SMPDB in 2009 (26). The goal of SMPDB was to provide internet accessible versions of high-quality, hand-drawn small-molecule pathways. While the results from the first version of SMPDB were visually appealing and the database proved to be popular within the target community, the use of multiple pathway artists led to problems with pathway consistency and quality. Furthermore, the reliance on static images and hand-annotated HTML image maps prevented pathways in the database from being updated or easily edited.

It was from the many frustrating experiences associated with constructing and maintaining the first version of SMPDB that we decided to develop PathWhiz. Essentially, PathWhiz is an attempt to combine the advantages of software-rendered pathway drawings with the aesthetic appeal of higher quality hand-drawn pathway diagrams. In effect, PathWhiz incorporates hard-coded SOPs into a pathway drawing program, thereby creating an environment for the generation of consistent and aesthetically appealing pathway drawings supported by rigorous data collection standards. While not yet perfect, we believe PathWhiz addresses many of the problems and shortcomings of our previous pathway drawing efforts. It also offers a number of important features and has some significant advantages over other pathway drawing tools. First, PathWhiz supports the generation of colourful, complex, visually pleasing and biologically rich pathway diagrams in a highly standardized way. Second, it allows for the simple and rapid generation of image-mapped pathway diagrams that can be easily zoomed and navigated. Third, it provides a framework for reading, writing and rendering pathway diagrams in machine-readable, easily exchanged formats (BioPAX, SBGN-ML, SBML, PWML). Fourth, as a web server, PathWhiz makes biological pathway generation far easier and pathway accessibility much greater because it is largely platform-independent and accessible from almost anywhere, anytime. Fifth, because of its web server design, PathWhiz supports community pathway contributions. This opens the door to a form of pathway crowd sourcing to more rapidly generate new pathways for database deposition and sharing.

Many different users have extensively tested PathWhiz during its development over the last few years. Throughout this testing period its operations have been optimized and its GUI streamlined through constant user feedback. As a result of these improvements, PathWhiz was recently used to generate more than 700 different human metabolomic pathways for version 2.0 of SMPDB (20), covering common metabolic pathways, metabolic disease pathways, signalling pathways, drug metabolism pathways, and drug action pathways. PathWhiz is currently being used to add to this very extensive collection and expand the content in SMPDB to new organisms.

Since it's inception 2 years ago, the PathWhiz studio has grown from 124 pathways in August 2013 to 722 pathways in August 2014, and currently contains a total of 819 pathways (166 metabolic, 12 physiological, 25 signalling, 320 drug action, 64 drug metabolism and 232 disease). It also houses 40,947 small-molecule compounds (40,732 of which are from HMDB, encompassing ∼96% of HMDB) and 9625 proteins (all of which can be mapped to UniProt). All of PathWhiz's data is available for download on the ‘Downloads’ page (http://smpdb.ca/pathwhiz/downloads). However, it is important to note that the elements in PathWhiz were (and are continually) added by users in response to user needs, thus the PathWhiz repository is not meant to serve as a primary resource for biological data. Instead, the PathWhiz repository is more of a pathway art studio rather than a pathway art gallery or database. An art studio typically has finished works or art, unfinished works of art as well as art ‘failures’, while a gallery only presents the best or most complete works of art.

Overall, we believe that the graphical tools and visualization capabilities of today's modern web browsers have moved well beyond the limitations associated with simplified wire-framed or schematic pathway diagrams. Indeed, many of these older pathway drawing tools and their associated pathway diagrams or databases were developed in the very earliest days of the internet. Given that more than a decade has passed since these early tools and earlier drawing conventions appeared, we believe the time is ripe for a change. A new generation of pathway drawing tools, a new generation of pathway databases and a new approach to generating, sharing and visualizing biological pathways on the internet need to be considered. By releasing PathWhiz as a public web server, we are hoping that it will inspire others to contribute additional pathways to the scientific community and to further enhance both PathWhiz's capabilities and the quality of biological pathways found on the internet.
